# High-Availability Computing Platform with Sensor Fault Resilience

**DOI:** 10.3390/s21020542

**Published:** 2021-01-13

**Authors:** Yen-Lin Lee, Shinta Nuraisya Arizky, Yu-Ren Chen, Deron Liang, Wei-Jen Wang

**Affiliations:** 1 Department of Computer Science and Information Engineering, National Central University, Taoyuan 320, Taiwan; yenlinlee811109@gmail.com (Y.-L.L.); shinta.nuraisya@gmail.com (S.N.A.); xaverchen@iii.org.tw (Y.-R.C.); deronliang@gmail.com (D.L.); 2 Institute for Information Industry, Taipei 106, Taiwan

**Keywords:** failover, high availability, sensor fault, fault detection and recovery, liveness detection

## Abstract

Modern computing platforms usually use multiple sensors to report system information. In order to achieve high availability (HA) for the platform, the sensors can be used to efficiently detect system faults that make a cloud service not live. However, a sensor may fail and disable HA protection. In this case, human intervention is needed, either to change the original fault model or to fix the sensor fault. Therefore, this study proposes an HA mechanism that can continuously provide HA to a cloud system based on dynamic fault model reconstruction. We have implemented the proposed HA mechanism on a four-layer OpenStack cloud system and tested the performance of the proposed mechanism for all possible sets of sensor faults. For each fault model, we inject possible system faults and measure the average fault detection time. The experimental result shows that the proposed mechanism can accurately detect and recover an injected system fault with disabled sensors. In addition, the system fault detection time increases as the number of sensor faults increases, until the HA mechanism is degraded to a one-system-fault model, which is the worst case as the system layer heartbeating.

## 1. Introduction

High availability (HA) [1] is an important feature of a cloud system, where availability is the percentage of time that an application or service is available at a given time [2,3]. From the perspective of cloud service providers, the availability of the cloud is a factor that affects customer choice, and this is not less important than the price [4]. For example, in February 2017, Amazon’s Simple Storage Service failed for 4 h and caused at least $150 million in losses to customers [5]. Therefore, in modern cloud services, there is a commitment between the service provider and the customer—that is, the Service-Level Agreements (SLAs) [6]—and HA is one of the important items in the agreement. In other words, modern cloud computing systems must protect the liveness of services/applications running on the computing pool of the system [7]. A common way to do this is to efficiently detect whether a system fault happens on a cloud service. Then, a corresponding fault recovery strategy is used to recover the cloud service in a short time.

A modern cloud computing system is comprised of a controller, a computing pool (physical hosts), virtual machines (VMs), a storage system, and a network infrastructure that connects the above components [8,9]. A cloud service usually runs on VMs, and the VMs are placed onto the physical machines of the computing pool. In order to ensure the HA of the cloud service, many studies have presented several HA techniques [9,10,11] for VM liveness detection and system fault recovery. For example, VMware vSphere [9] uses the heartbeat of the datastore, Internet Control Message Protocol (ICMP) [12], and VM I/O monitors to detect different types of system faults. Another HA example is presented in the paper by Tajiki et al. [13], which addresses the issue of efficient system fault recovery and system fault prevention for fog-supported Software Defined Networks (SDN). System fault recovery is triggered by the liveness detection of Fog Nodes, while system fault prevention is achieved by regular network topology reconfiguration. The scheme reroutes the network flow to recover and prevent system faults, optimize the energy consumption of the Fog Node and the reliability of the selected path and guarantee the required quality of service level.

**Figure 1:**
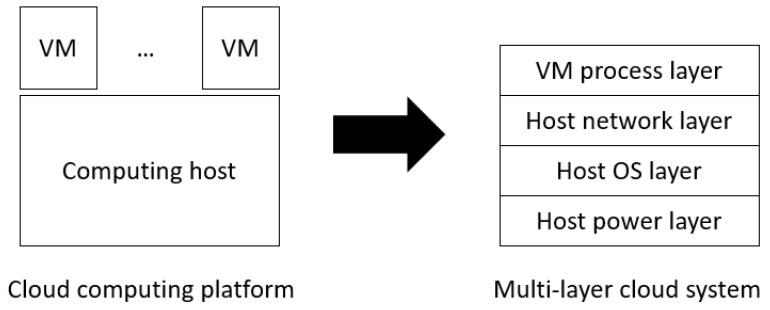
Schematic diagram of a multi-layer system.

According to prior studies [14,15,16,17], a cloud computing system can be abstracted into multiple layers, where the functionality of the upper layer depends on that of the lower layers [18,19]. For example, the host OS layer depends on the host power layer, the host network layer depends on the lower two layers, and the VM process layer depends on the lower three layers, as shown in Figure 1. This multi-layer abstraction facilitates the use of multi-sensor-based fault detection and may reduce system downtime [14,15]. However, one issue remains unsolved in multi-sensor-based fault detection: a fault may occur at any sensor and consequently disable the system fault detection procedure, meaning that the HA mechanism cannot identify and recover a system fault in the cloud service. To this end, this paper proposes a multi-sensor-based HA mechanism to continuously provide system (liveness) fault detection and recovery for a protected cloud service, even if some sensors become faulty.

In the proposed approach, a sensor is installed at each layer to report the liveness of the layer. The sensors can be implemented as a hardware-assisted component such as the Intelligent Platform Management Interface (IPMI) tool [20] or a software detection process such as an ICMP-based heartbeating component. Through these sensors, we develop the system fault detection method based on the Software-Defined High Availability Cluster (SDHAC) approach [14] to quickly determine whether a layer is faulty and choose an efficient recovery strategy to recover the protection target. The major problem of the original HA mechanism in the SDHAC is that a sensor can fail at any time, and then the HA mechanism designed based on the fault model using all of the sensors cannot work correctly. Typically, a sensor fault can be detected by checking its return format, values, and liveness (the ability to respond to a query in a given time). When a sensor fault has been detected, we propose the use of a dynamic fault model switching method that is able to reconstruct a new fault model with N−1 healthy sensors from the original fault model with *N* sensors. The system fault detection and recovery methods are updated based on the new fault model without human intervention. As a result, the multi-layer cloud system can continue providing HA protection for a cloud service on VMs.

To the best of our knowledge, this study is the first to address the sensor fault issue on cloud HA. With the assumption of the liveness of the sensor at the highest layer, the proposed HA mechanism can tolerate any detectable sensor fault and continue providing HA protection for the cloud computing system. To show how a sensor fault affects the system fault detection efficiency of the proposed HA mechanism, we have conducted several experiments on a four-layer cloud computing system by injecting different kinds of sensor faults and system faults. When a sensor fault is detected, the proposed mechanism attempts to automatically reduce the four-layer fault model to a three-layer fault model and continue to provide HA protection to the system. The fault model can be further reduced to a one-layer fault model with the sensor at the highest layer. For each fault model, we injected the same set of system faults to evaluate their system fault detection efficiency. According to the experimental results, the proposed mechanism can tolerate sensor faults, and the system fault detection efficiency gradually reduces as the number of healthy sensors decreases. Although, in some cases, the weighted (system) fault detection time of the proposed mechanism increases, it is still shorter than or equal to that of the traditional system layer heartbeating [21], which is a common system fault detection method that requires at least 30 s for system fault detection [9].

The remainder of this paper is organized as follows. Section 2 introduces related work. Section 3 illustrates the proposed mechanism through examples. Section 4 presents the experiment results and analysis. Section 5 presents our conclusions.

## 2. Related Work

In the survey papers [22,23], several fault detection and diagnosis techniques have been studied. Although most of those techniques utilize multiple sensors, they are designed to accurately identify whether a fault is happening based on several raw signals from the sensors. For example, many practical systems such as vehicles [24], aircrafts [25], and manufacturing systems [26] use multiple sensors to perform fault detection and diagnosis for different critical components. These kinds of techniques are not applicable to liveness detection for cloud services. The most popular technique for liveness detection in the cloud is heartbeating [21]. In this technique, the detection target has to send at least one live message to the liveness detector (sensor) regularly, typically every 30 s to 2 min [9]. In this section, we introduce related studies [9,14,15,19] that use multiple sensors to monitor or detect whether a cloud service/application is live or not. None of these studies have discussed continuous fault detection with sensor faults in the cloud.

### 2.1. Multi-Sensor-Based Monitoring in the Cloud

Trihinas et al. [19] use multiple sensors to monitor the performance and behavior of cloud services and their underlying infrastructure. The proposed monitoring system aims to monitor elastically adaptive multi-cloud services. In their system, the following three cases must be considered: (i) resource allocation and application tuning, (ii) network and security problems detection, and (iii) verification on the Service-Level Agreements. Therefore, the authors proposed the monitoring of several heterogeneous components, ranging from low-level system components such as the CPU, memory, disk, and network to high-level system components such as application throughput and request latency. As the system only supports monitoring in the cloud, it does not include any HA design for cloud services running on the platform. Many existing cloud monitoring tools [27,28,29] also provide similar functionalities to support multi-layer monitoring.

### 2.2. NCU-HA and Software-Defined High Availability Cluster (SDHAC)

The NCU-HA [15] aims to provide system (liveness) fault detection and recovery mechanism for the open source VM project QEMU-KVM [30]. The NCU-HA can be run on both low-cost personal computers (PCs) and Intelligent Platform Management Interface (IPMI)-based servers. The NCU-HA can use various detectors (IPMI, ICMP-based sensors and Libvirt [31]) on the host to determine the type of system fault and recover from it.

The SDHAC [14] was developed based on the concept of NCU-HA. The goal for SDHAC is to provide system fault detection and a recovery mechanism for virtual machines (VMs) on the computing pool at the OpenStack platform [8]. The SDHAC can detect a system fault by using various sensors (IPMI, ICMP-based sensors, and Libvirt) in a sequential manner, from top-layer system sensors to low-layer system sensors. Then, it performs the corresponding recovery methods to recover the failed VMs and the faulty nodes.

### 2.3. Cloud Platform HA

In VMWare vSphere [9], when a host is added to a vSphere HA cluster, an agent (sensors) is installed on the host. Each host in the cluster acts as a primary host or a secondary host, where the primary host communicates with vCenter Server and monitors the status of all protected VMs and secondary hosts. In a cluster, there is only one primary host at a time. For host protection, the primary host monitors the liveness of the secondary hosts through a network heartbeat sensor, which exchanges liveness signals every second. When the primary host loses the network heartbeats from a secondary host within a set time, it then uses another sensor to check the exchange heartbeat between the secondary host and one of the datastores. If the secondary host is not exchanging heartbeats with any datastore, the secondary host is considered to be faulty. Then, the VMs on the secondary host are restarted on alternate hosts. If the secondary host is exchanging heartbeats with a datastore, it is considered that the secondary host is located in a network partition or isolated from the network. In this case, the primary host only keep monitoring the VMs on the secondary host. If the VM to be restarted is in the power-off state, the primary host places the VM onto another normal host. For VM protection, when the heartbeat signal from the protected VM is not received by the primary host within a set time, the primary host determines that the VM has failed, and then the VM is rebooted to recover the VM. The set time ranges from 30 s to 2 min. It is worth mentioning that the focus of VMware vSphere HA is on system fault detection and recovery, and thus the handling strategy of sensor faults is not mentioned.

On the other hand, OpenStack [8] recommends that users apply Pacemaker-based HA techniques in their environment, where Pacemaker [32] is an open source HA resource manager based on heartbeating. Most HA techniques for OpenStack use heartbeating-based sensors to detect the liveness faults in the VMs and the computing hosts. They handle VM-level and host-level faults by evacuating and rebooting failed VMs, respectively. However, none of these techniques consider sensor faults. Specifically, many HA techniques treat the fault of a heartbeating-based sensor as a target service fault. This is because the heartbeating-based sensor is robust, and its unexpected response or unresponsiveness is mainly caused by the fault of the target service.

## 3. The Proposed Mechanism

In this section, we first define the symbols used in the section. Then, we use the symbols to explain the concept of multi-layer systems and their fault models with *N* sensors. Consequently, we explain the proposed mechanism that can handle sensor faults with the proposed fault model reconstruction method, and then use an example to illustrate fault model reconstruction.

### 3.1. Symbols

Table 1 defines the symbols that are used in this section.

### 3.2. The Multi-Layer Cloud System

**Figure 2:**
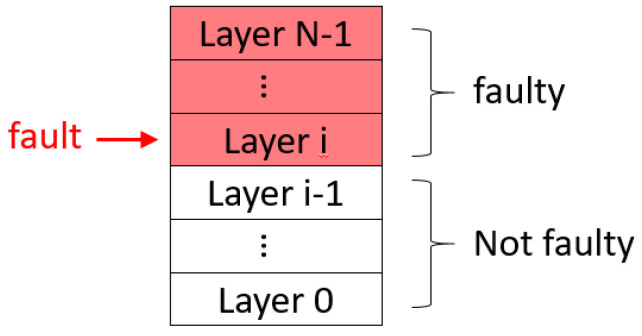
The impact of a system fault on a multi-layer system with the layer dependency property.

A modern cloud computing system can be abstracted into a multi-layer system. As shown in Figure 1, a cloud computing system is divided into a four-layer system, which consists of a host power layer, a host operating system (OS) layer, a host network layer, and a virtual machine (VM) process layer. As the multi-layer is an abstracted concept of the system, the system can be partitioned into N layers, depending on the property of the subcomponents of the system and the sensors used for these subcomponents, as shown in Figure 2. In the N-layer system, we install a sensor for each layer, that is, $D_{0}$ to $D_{N-1}$. For liveness detection, a system must be able to handle transient faults that could occur and then be recovered automatically before the system is considered not to be live. As a result, each layer has at most two kinds of faults, as described below.

- Transient fault: A system fault with a limited duration, which will temporarily hinder the normal operation of the system until the system fault disappears [33], such as the network being busy. It is worth mentioning that the system can still operate normally after the transient fault disappears.
- Permanent fault: The system fault will permanently hinder the normal operation of the system until the system fault is eliminated manually or by a system, such as host power off.

In a multi-layer system, there is an important and useful property called (liveness) layer dependency. This property has been reported in several prior studies. For example, Tchana et al. [16] indicated that the hardware layer fault can trigger faults at high layers (VM and application) in a three-layer system. Here, we propose a layer dependency property for N layers as follows.

- Liveness layer dependency: in a system, the liveness of the upper layer depends on the liveness of the lower layers.

This means that a system fault in a lower layer can result in the failure of the liveness of all upper layers, but a system fault in an upper layer cannot impede the liveness of any lower layer. For example, Figure 2 is an N-layer system. When a system fault occurs at the layer $L_{i}$ (0 ≤ i ≤N−1), all the layers above $L_{i}$ must fail. On the contrary, all the layers below the $L_{i}$ do not fail. An example in a real system: When the host network fails, the user cannot access the VM, but the host power and OS are still live. Similarly, the network being busy (transient fault) causes the VM to be temporarily inaccessible until the network becomes live again, and the VM will not be damaged after the network stops being busy.

### 3.3. Continuous Liveness Detection and Fault Recovery with Sensor Faults

In this study, we assume that the highest-layer sensor never fails. There are two reasons for this: first, the faults at the highest-layer sensor result in incomplete system fault detection, which is beyond our research scope, and second, the highest-layer sensor is usually the most robust in practical cloud systems. As liveness detection on the highest layer usually requires a long time to distinguish transient faults from permanent faults, we can use other low-layer sensors to accelerate this process. In addition, we assume that only one system fault in the cloud system occurs at a time, due to problem simplification. This means that no other system faults can occur in the same host of the computing pool until the protected target has been recovered.

**Figure 3:**
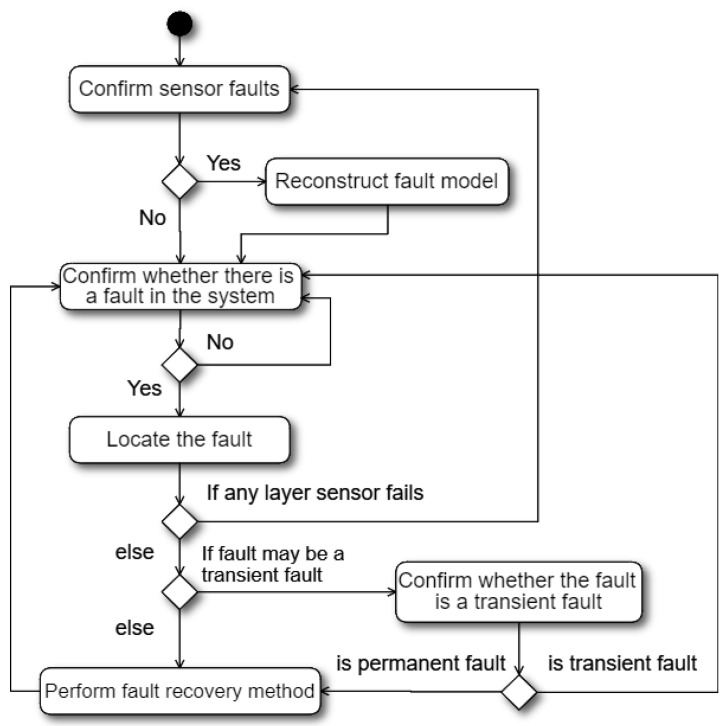
Flow chart of the proposed high-availability mechanism.

Based on the layer dependency feature of the multi-layer system, we propose a new HA mechanism based on fault model reconstruction, as shown in Figure 3. The mechanism first checks sensor faults, and then reconstructs the fault model based on the detected sensor faults if needed. Then, the mechanism confirms whether there is a system fault in the cloud system by using the sensor of the highest layer. This is because all faults in the system can be detected by the sensor of the highest layer according to the liveness layer dependency. If the highest layer sensor detects a system fault, the mechanism will detect the remaining layers according to the fault model to find the system fault location. The common method of locating the system fault is to detect the remaining layers in order from highest to lowest until the lowest faulty layer is found, where the lowest faulty layer is the location of the root cause. If any sensor fault occurs while finding the system fault, the mechanism needs to reconstruct the fault model and then restarts the new system fault detection process based on the newly constructed fault model. The method for identifying sensor faults can be achieved by checking the return format, values, and liveness (the ability to respond to a query in a given time) of the sensor. On the contrary, if there is no sensor fault and the system fault location (the lowest faulty layer) is found, the mechanism directly checks whether the system fault location may have a transient fault according to the fault model. If the system fault location may have a transient fault, the mechanism continuously uses the sensor at the lowest faulty layer within the transient fault detection time to confirm whether the fault is transient. If the mechanism has confirmed that the system fault is a transient fault because the layer has responded to the liveness query of its layer sensor, the mechanism simply ignores the transient fault and begins the next round of system fault detection. On the contrary, if the mechanism has confirmed that the system fault is a permanent fault, the mechanism performs the corresponding fault recovery method to recover the system. After the failed system is successfully recovered, the mechanism then starts the next round of system fault detection.

### 3.4. Fault Model Reconstruction and Switching

The fault model reconstruction method (Algorithm 1) is described as follows.
**Algorithm 1** fault_model_reconstruction**Input:** the faulty sensor $D_{i}$ and the corresponding layer $L_{i}$.**Output:** a fault model. 1:If $L_{i}$ is the highest layer, return Reconstruction_Failure. 2:Combine the layers $L_{i}$ and $L_{i+1}$ into a new layer $L_{i}^{\prime }$. 3:Use the sensor $D_{i+1}$ as the sensor $D_{i}^{\prime }$ of $L_{i}^{\prime }$. 4:Calculate the value Tx as the maximum of $T_{to}\left(D_{i}\right)$ and $T_{to}\left(D_{i+1}\right)$. Update $T_{to}\left(D_{i}^{\prime }\right)$ to be the smallest multiple of $T_{sr}\left(D_{i+1}\right)$ that is larger than or equal to Tx. The total sensor detection time can be divided into a sensor response time and a transient fault detection time. 5:Update $T_{sr}\left(D_{i}^{\prime }\right)$ with $T_{sr}\left(D_{i+1}\right)$. 6:Update $T_{td}\left(D_{i}^{\prime }\right)$ with the difference between the total sensor detection time and its sensor response time. 7:If $T_{td}\left(D_{i}^{\prime }\right)$ is larger than 0, $L_{i}^{\prime }$ includes transient faults. 8:Reuse $R_{i}$ as the recovery method of $L_{i}^{\prime }$. 9:For k=0∼i−1, reuse the system fault detection and recovery methods of $L_{k}$ at $L_{k}^{\prime }$.10:For k=i+2∼N−1, reuse the system fault detection and recovery methods of $L_{k}$ at $L_{k-1}^{\prime }$.11:**return** the new fault model based on the new multi-layer system from $L_{0}^{\prime }$ to $L_{N-2}^{\prime }$.

The concept of Algorithm 1 is to merge the layer $L_{i}$ for which the sensor $D_{i}$ has failed with the normal layer $L_{i+1}$, as described in Step 2. Then, the new merged layer, namely, $L_{i}^{\prime }$, needs to be updated with new system fault detection and recovery methods. In the case that $T_{to}\left(D_{i}^{\prime }\right)$ > $T_{sr}\left(D_{i}^{\prime }\right)$, the proposed HA mechanism needs to wait for a sufficient time to cover transient faults, as shown in Steps 4 to 7. Then, the mechanism directly applies the recovery method for $L_{i}$ to the new layer $L_{i}^{\prime }$ because the low-layer recovery method must guarantee the recovery of the upper layers, as shown in Step 8. Take Figure 1 as an example: the recovery method of the host OS layer needs to consider the recovery of the protection target—the VM process—which means the above layers must be healthy after recovery. A typical solution for this case is to evacuate the VM to a healthy host. Other layers are not affected by the proposed fault model reconstruction method and can be reused, as shown in Steps 9 and 10.

To analyze the computational and space complexity, we assume that the cloud computing pool consists of *M* hosts and each host has *N* layers. The proposed mechanism uses sequential detection to scan whether a system fault exists at some layer, which is similar to the system fault detection method of the SDHAC [14], and creates *K* threads to perform parallel liveness detection for each host. The computational complexity of the proposed fault model reconstruction method is O(N), and the space complexity is O(M∗N) for a sensor fault; the computational complexity of the system fault detection method is $O(\frac{M}{K}+N)$, and the space complexity is O(M∗N) for each system fault at the cloud system. When the number of faulty hosts is *L*, only the computational complexity of the system fault detection method changes to $O(\frac{M-L}{K}+\frac{L}{K}*N)$. Generally, *N* and *L* are small, and *M* is much larger than *N* and *L*. Therefore, the computational complexity of the system fault detection method is close to $O(\frac{M}{K})$.

It is worth mentioning that the number of possible fault models is $2^{N-1}$. Although increasing *N* could help the HA mechanism to achieve faster system fault detection [14] or faster recovery [8,9,14,15], it could greatly complicate the design and implementation of the HA mechanism because more system fault detection sensors and system fault recovery methods would need to be included. As a result, N should be a small number in practice.

### 3.5. Example of Fault Model Reconstruction

After reconstructing the fault model, the proposed mechanism should switch the fault model and continue to detect system faults based on the new fault model. In the following, we employ a seven-layer (*N* = 7) system comprised of $L_{0}$ to $L_{6}$ layers as an example to explain the details of how to reconstruct the fault model.

First of all, the fault model used in this study can be shown in two important tables: the fault-symptom table [34,35] and the sensor information table, as shown in Table 2 and Table 3. Table 2 is the fault-symptom table of the seven-layer system, which is used to list the symptoms of all system faults in the cloud system. In the fault-symptom table, the system fault location is a layer and the system fault symptom is a list. The elements in the system fault symptom list represent the results detected by all sensors from $D_{0}$ to $D_{N-1}$ when a system fault occurs, where 0 means that the sensor detects that the layer is not faulty, and 1 means that the sensor detects that the layer is faulty. For example, when a system fault occurs at $L_{1}$, only the detection result of $D_{0}$ is 0; this is because all other layers except $L_{0}$ are faulty.

Table 3 is the sensor information table, which lists the information of all sensors used in the seven-layer system. For each sensor, the table displays the sensor ID, the layer to be detected by the sensor, the value of the “Including transient faults?” column, the total sensor detection time of the layer sensor and the sensor response time of the layer sensor. The “Including transient faults?” column shows whether the layer may generate a transient fault. The total sensor detection time is the sum of the sensor response time and the transient fault detection time.

In order to facilitate the explanation of the reconstruction method of the fault model, the definition of the new layer type is given below.

- Active layer: The layer in which the layer sensor is working.
- Disabled layer: The layer in which the layer sensor has failed.

According to the status of the layer sensor, the layers are divided into active layers and disabled layers. According to the liveness layer dependency, we can detect system faults in the disabled layer through the sensor of the active layer higher than the disabled layer. Therefore, we first reorganize the layer by merging the disabled layer and the higher active layer as a new layer called the combination layer.

- Combination layer: The layer consists of an active layer and several continuous lower disabled layers and uses the sensor of the active layer as its layer sensor.

**Figure 4:**
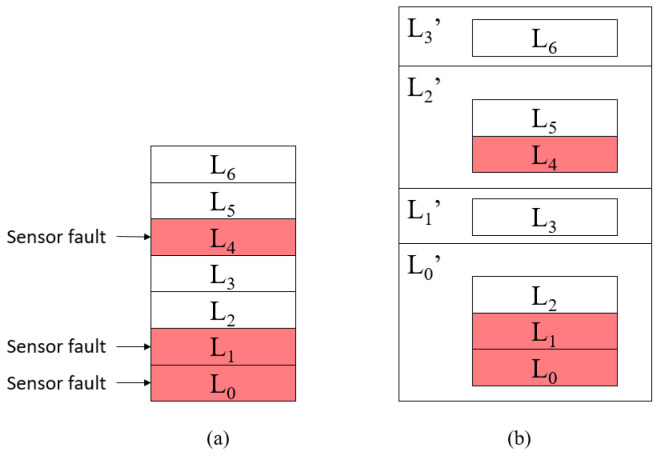
(**a**) Seven-layer system with three disabled layers. (**b**) The four-layer system generated by the fault model reconstruction method.

In Figure 4, Figure 4a is a seven-layer system in which the sensors $D_{0}$, $D_{1}$, and $D_{4}$ have failed. Then, we merge $L_{0}$, $L_{1}$, and $L_{2}$ into one combination layer $L_{0}^{\prime }$ and merge $L_{4}$ and $L_{5}$ into another combination layer $L_{2}^{\prime }$ based on the proposed fault model reconstruction method, one at a time. The final result is shown in Figure 4b. Thus, we can detect the system faults of $L_{0}$, $L_{1}$, and $L_{2}$ through $D_{2}$ and detect the system faults of $L_{4}$ and $L_{5}$ through $D_{5}$. After that, we obtain a new layer list, which contains $L_{0}^{\prime }$, $L_{1}^{\prime }$, $L_{2}^{\prime }$, and $L_{3}^{\prime }$. This means that the seven-layer system becomes a four-layer system. Moreover, the layers in the new layer list still have liveness layer dependency.

Next, we can update the fault-symptom table based on the active sensors and liveness layer dependency, as shown in Table 4. We use sensor $D_{2}$ as sensor $D_{0}^{\prime }$, sensor $D_{5}$ as sensor $D_{2}^{\prime }$, and reuse sensors $D_{3}$ and $D_{6}$ as sensors $D_{1}^{\prime }$ and $D_{3}^{\prime }$, respectively. In Table 4, the elements of the system fault symptom list represent the detection results of $D_{0}^{\prime }$, $D_{1}^{\prime }$, $D_{2}^{\prime }$, and $D_{3}^{\prime }$, respectively. Similarly, we must also update the sensor information table, as shown in Table 5. We first update the sensor list, sensor ID list, and detection target list; then, we use the fault model reconstruction to obtain the corresponding fault model parameters, such as the total sensor detection time, sensor response time, the need to handle transient faults, and the recovery method. Note that the sensor information table only records the necessary parameters for system fault detection.

In order to recover the protected target, the proposed fault model reconstruction method must be able to automatically create new recovery methods for $L_{0}^{\prime }$ and $L_{2}^{\prime }$. The new recovery method $R_{0}^{\prime }$ is equal to $R_{0}$. Similarly, $R_{2}^{\prime }$ is equal to $R_{4}$. Finally, the proposed fault model reconstruction method reuses $R_{3}$ and $R_{6}$ as $R_{1}^{\prime }$ and $R_{3}^{\prime }$, respectively.

## 4. Experimental Results

### 4.1. Experimental Environment

**Figure 5:**
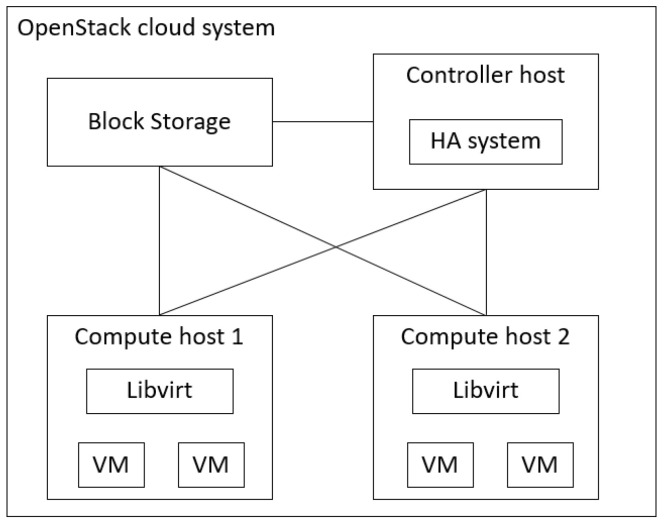
Architecture diagram of the experimental environment.

The proposed fault model reconstruction method can be used in a cloud system of N layers. The goal of our experiment was to evaluate the impact of fault model reconstruction on the performance of the HA mechanism. To do this, we injected sensor faults to the system in turn and then observed how the ability of system fault detection was affected. We implemented an HA mechanism for a four-layer OpenStack cloud system based on the proposed mechanism, as shown in Figure 5. The fault model for the four-layer system could include another seven fault models. The details of the experiment are shown in Section 4.3. The OpenStack version we applied was Queens. In the OpenStack system, there is a block storage host, a controller host, and several compute hosts. On each compute host, we placed several virtual machines (VMs). The specifications of the hosts are shown in Table 6. We set up all VMs with two vCPUs, 2 GB of memory, 25 GB disks, and an Ubuntu server 16.04 as the operating system (OS).

We abstracted the OpenStack cloud system into a four-layer system, where the lower to higher layers were the host power, the host OS, the host network layer, and the VM process layers. The fault-symptom table and the sensor information table are shown in Table 7 and Table 8. We used the Intelligent Platform Management Interface (IPMI) to detect the host power layer and the host OS layer, the ICMP-based sensor to detect the host network layer, and Libvirt as a sensor to detect the liveness of VM processes. Although transient faults may occur in the host OS layer and the VM process layer, we did not divide the total sensor detection time of these two layer sensors into sensor response time and transient fault detection time, because the total sensor detection time of these two layer sensors was very short. It is worth mentioning that the actual sensor response time may have been shorter than the given sensor response time listed in Table 8; this is because the sensor response time is the deadline to respond to liveness query.

### 4.2. Sensor Information

To detect sensor faults, the correctness of the sensor information should be discussed. The IPMI sensor includes IPMItool software and IPMI-compliant hardware. The response of the IPMI sensor and the Watchdog of IPMI sensor is in the form of a message with a specific format, which contains the host power status and OS countdown timer value, respectively. The ICMP-based sensor and Libvirt are software components that run on the computing host, and their status can be detected by querying the host OS. The response of the ICMP-based sensor and Libvirt is in the form of a message with a specific format, which contains time-to-live message of the network packet and the VM process status, respectively. Therefore, the HA system can detect the faults of the first two sensors by checking whether the response is received and the correctness of the response format, and it can detect the faults of the latter two sensors by checking the correctness of the response format and the sensor status.

### 4.3. Experimental Design

In the experiments, we disabled different sets of sensors in the proposed HA mechanism in order to observe the system fault detection efficiency based on different fault models. In total, seven possible fault models could be created by the proposed fault model reconstruction method, along with the original fault model with four active sensors, as shown in Table 9. System fault detection based on different fault models can have different results, because the fault models that use fewer sensors may have worse detection efficiency. To evaluate the system fault detection performance of the fault models, we injected system faults into the OpenStack system according to the system faults of each layer. The method of injecting system faults is shown in Table 10. The host power fault involved turning off the power of the host, the host OS fault involved making the host OS hang, the host network fault involved making the host inaccessible through the network, and the VM process fault involved forcing the VM to crash.

### 4.4. Experimental Results

In order to evaluate the performance and correctness of the proposed HA mechanism in different fault models, we injected each system fault at least 10 times to obtain the average system fault detection time and confirm whether the system had properly recovered. The system fault detection time was the time from the start of the system fault injection to the determination of the system fault type. Table 11 shows the average time for the HA mechanism to detect different system faults based on different fault models, where the time unit is seconds.

Here, we used a common system fault detection method—the system layer heartbeating—as the comparison baseline. The system layer heartbeating can be regarded as a sensor used in a one-layer system. This is because the system layer heartbeating usually only detects the liveness of the protection target, that is, the highest layer. The system layer heartbeating used in cloud systems usually spends at least 30 s on system fault detection, in particular for transient network faults. In this study, we set the system layer heartbeating time to 31 s.

**Figure 6:**
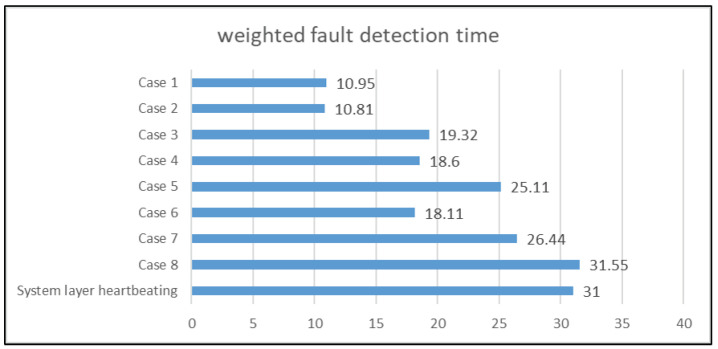
Comparison of weighted fault detection time (in seconds).

For comparison, the weighted system fault detection time of each fault model and system layer heartbeating was needed. To calculate the weighted fault detection time of different fault models, we needed to multiply the system fault probability by the average system fault detection time for each system fault type and then add them together. The system fault probability values used in this study were based on the National Center for Supercomputing Applications (NCSA) Platinum and Titan fault data summary [36]. As the software fault included the system fault of the VM process, the host network, and the host OS layers, the system fault probability of these three layers was 26.2%. In addition, as the host OS layer could detect hardware system faults, the system fault probability of the host OS layer was 26.2 + 2.84 = 29.04 (%). In summary, the system fault probabilities from the VM process layer to the host power layer were 26.2%, 26.2%, 29.04%, and 18.56%, respectively. The results are shown in Figure 6.

**Figure 7:**
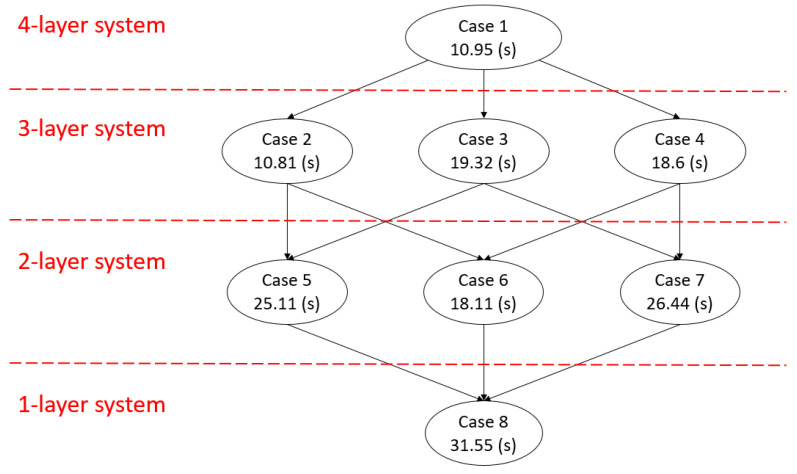
The weighted average of system fault detection time for all cases and the fault model switching paths.

According to the experimental results, we know that the HA mechanism can always tolerate sensor faults and can accurately recover the failed protected target. Figure 7 shows the weighted system fault detection time of each case and the relationship between the cases. When a sensor fails, the proposed HA mechanism will lose part of its detection ability, thereby increasing the system fault detection time. Although in some cases, the proposed mechanism requires a longer weighted fault detection time, the time is still less than or equal to the weighted fault detection time of the system layer heartbeating. Generally, in terms of detection efficiency, the original fault model that has not been reconstructed has the best system fault detection efficiency. As the number of reconstructions increases, the performance of the reconstructed fault model decreases. Finally, the reconstructed fault model is degraded to a one-layer model, which is the worst case and has the same performance as the system layer heartbeating. In this experiment, the performance (weighted fault detection time) of the original fault model is 10.95 s, the performance of the fault models with one failed sensor ranges from 10.81 to 19.32 s, the performance of the fault models with two failed sensors ranges from 18.11 to 26.44 s, and the performance of the fault models with three failed sensors is 31.55 s. As each sensor has different system fault detection ability, the loss of different sensors has different effects on the system fault detection time. As a result, the weighted system fault detection time does not decrease linearly in terms of the number of sensor faults.

## 5. Conclusions

In this study, we proposed a HA mechanism that can dynamically change the fault model in response to different sensor faults. We implemented the proposed HA mechanism on a four-layer OpenStack cloud platform. According to the experimental results, for all possible sensor faults, the proposed mechanism can correctly detect all types of system faults and recover the protected target form the system faults. In addition, according to our experimental results, the average of weighted system fault detection time for zero-sensor fault, one-sensor fault, two-sensor faults, and three-sensor faults are 10.95 s, 16.24 s, 23.22 s, and 31.55 s, respectively. Those results are better or equal to the system fault detection time (31 s) for system layer heartbeating. The experiment results (Figure 7) also show that as the number of reconstructions increases, the performance of the reconstructed fault model decreases. This is because the system fault detection capability of the fault model is reduced due to the sensor faults. Eventually, the model can be degraded to one-layer system with only the ability to perform system layer heartbeating.

## Figures and Tables

**Table 1:** Definition of symbols.

Symbol	Description
N	The cloud system is abstracted into N layers
$L_{i}$	The *i*-th layer of the cloud system, (0 ≤ i ≤ N − 1)
$D_{i}$	The sensor used to detect $L_{i}$, (0 ≤ i ≤ N − 1)
$R_{i}$	The system fault recovery method used to recover $L_{i}$, (0 ≤ i ≤ N − 1)
$T_{sr}\left(D_{i}\right)$	The sensor response time of sensor $D_{i}$. (0 ≤ i ≤ N − 1)
$T_{td}\left(D_{i}\right)$	The transient fault detection time of sensor $D_{i}$. (0 ≤ i ≤ N − 1)
$T_{to}\left(D_{i}\right)$	The total sensor detection time of sensor $D_{i}$, where $T_{to}\left(D_{i}\right)$ = $T_{sr}\left(D_{i}\right)$ + $T_{td}\left(D_{i}\right)$

**Table 2:** Fault-symptom table of a seven-layer cloud system used for the fault detection method.

System Fault Location	Symptoms Detected by All Sensors from $D_{0}$ to $D_{6}$
	[$D_{0}$, $D_{1}$, $D_{2}$, $D_{3}$, $D_{4}$, $D_{5}$, $D_{6}$]
$L_{0}$	[1, 1, 1, 1, 1, 1, 1]
$L_{1}$	[0, 1, 1, 1, 1, 1, 1]
$L_{2}$	[0, 0, 1, 1, 1, 1, 1]
$L_{3}$	[0, 0, 0, 1, 1, 1, 1]
$L_{4}$	[0, 0, 0, 0, 1, 1, 1]
$L_{5}$	[0, 0, 0, 0, 0, 1, 1]
$L_{6}$	[0, 0, 0, 0, 0, 0, 1]

**Table 3:** Layer sensor and corresponding detection information of a seven-layer system.

Sensor Name	ID	Detection Target (Layer)	Including Transient Faults?	Total Sensor Detection Time (s)	Sensor Response Time (s)
$D_{0}$	0	$L_{0}$	N	1	1
$D_{1}$	1	$L_{1}$	Y	4	2
$D_{2}$	2	$L_{2}$	N	2	2
$D_{3}$	3	$L_{3}$	Y	30	1
$D_{4}$	4	$L_{4}$	N	3	3
$D_{5}$	5	$L_{5}$	N	2	2
$D_{6}$	6	$L_{6}$	N	1	1

**Table 4:** The updated fault-symptom table of the new four-layer system.

System Fault Location	Symptoms Detected by All Sensors from $D_{0}$’ to $D_{3}$’
	[$D_{0}^{\prime }$, $D_{1}^{\prime }$, $D_{2}^{\prime }$, $D_{3}^{\prime }$]
$L_{0}^{\prime }$	[1, 1, 1, 1]
$L_{1}^{\prime }$	[0, 1, 1, 1]
$L_{2}^{\prime }$	[0, 0, 1, 1]
$L_{3}^{\prime }$	[0, 0, 0, 1]

**Table 5:** The updated sensor information table of the new four-layer system.

Sensor Name	ID	Detection Target (Layer)	Including Transient Faults?	Total Sensor Detection Time (s)	Sensor Response Time (s)
$D_{0}^{\prime }$	2	$L_{0}^{\prime }$	Y	4	2
$D_{1}^{\prime }$	3	$L_{1}^{\prime }$	Y	30	1
$D_{2}^{\prime }$	5	$L_{2}^{\prime }$	Y	4	2
$D_{3}^{\prime }$	6	$L_{3}^{\prime }$	N	1	1

**Table 6:** Specification table of the hosts in the system to be tested.

Host	Operating System	CPU	Memory	Disks
Controller	Ubuntu Server 16.04	Intel Xeon E5-2620v2	48 GB	300 GB
Compute 1	Ubuntu Server 16.04	Intel Xeon E5-2620v2	48 GB	500 GB
Compute 2	Ubuntu Server 16.04	Intel Xeon E3-1240v5	16 GB	1 TB
Block Storage	Ubuntu Server 16.04	Intel^®^ Core™ i7-3770	8 GB	1 TB

**Table 7:** Fault-symptom table of the system under testing. VM: virtual machine.

System Fault Location	System Fault Symptom
Host power layer	[1,1,1,1]
Host OS layer	[0,1,1,1]
Host network layer	[0,0,1,1]
VM process layer	[0,0,0,1]

**Table 8:** Layer sensor and corresponding detection information. IPMI: Intelligent Platform Management Interface. ICMP: Internet Control Message Protocol.

Sensor Name	ID	Detection Target (Layer)	Including Transient Faults?	Total Sensor Detection Time (s)	Sensor Response Time (s)
IPMI	0	host power	N	0.06	0.06
Watchdog of IPMI	1	host OS	Y	4	4
ICMP-based sensor	2	host network	Y	31	1
Libvirt	3	VM process	Y	1	1

**Table 9:** Fault models with the IDs of active sensors.

Fault Model Cases	IDs of Active Sensors
1	3,2,1,0
2	3,2,1
3	3,2,0
4	3,1,0
5	3,2
6	3,1
7	3,0
8	3

**Table 10:** System fault injection method.

System Fault Type	Description	System Fault Injection Method
Host power fault	Compute node loses power	Power off the compute node
Host OS fault	Compute node OS not responding	Kill the fist process in the compute node
Host network fault	Compute node cannot be connected	Shut down the network card of the compute node
VM process fault	VM process is not running	Terminate the VM process in the compute node

**Table 11:** Detection time of various system faults in each fault model case (in seconds).

Fault Model Cases	VM Process Fails	Host Network Isolation	Host OS Hangs	Host Power Off
1	1.99	32.78	3.78	4.02
2	2.02	32.76	3.52	3.62
3	2.82	32.17	32.64	3.62
4	31.55	33.88	2.87	3.15
5	2.77	33.26	32.46	33.64
6	31.44	32.37	2.93	2.94
7	32.19	31.57	31.68	2.88
8	31.56	31.8	31.21	31.72

## References

[1] Mesbahi M.R., Rahmani A.M., Hosseinzadeh M. (2018). Reliability and high availability in cloud computing environments: A reference roadmap. Hum.-Centric Comput. Inf. Sci..

[2] Nabi M., Toeroe M., Khendek F. (2016). Availability in the cloud: State of the art. J. Netw. Comput. Appl..

[3] Endo P.T., Rodrigues M., Gonçalves G.E., Kelner J., Sadok D.H., Curescu C. (2016). High availability in clouds: Systematic review and research challenges. J. Cloud Comput..

[4] Armbrust M., Fox A., Griffith R., Joseph A.D., Katz R., Konwinski A., Lee G., Patterson D., Rabkin A., Stoica I. (2010). A View of Cloud Computing. Commun. ACM.

[5] Condliffe J. Amazon’s $150 Million Typo Is a Lightning Rod for a Big Cloud Problem. https://www.technologyreview.com/2017/03/03/153431/amazons-150-million-typo-is-a-lightning-rod-for-a-big-cloud-problem/.

[6] Amazon AWS Service Level Agreements (SLAs). https://aws.amazon.com/legal/service-level-agreements/.

[7] Wang W.-J., Huang H.-L., Chuang S.-H., Chen S.-J., Kao C.H., Liang D. Virtual machines of high availability using hardware-assisted failure detection. Proceedings of the International Carnahan Conference on Security Technology (ICCST).

[8] OpenStack OpenStack Documentation. https://docs.openstack.org/queens/index.html.

[9] VMware vSphere Availability. https://docs.vmware.com/en/VMware-vSphere/6.5/vsphere-esxi-vcenter-server-65-availability-guide.pdf.

[10] Wang T., Zhang W., Wei J., Zhong H. Fault detection for cloud computing systems with correlation analysis. Proceedings of the 2015 IFIP/IEEE International Symposium on Integrated Network Management (IM).

[11] Fan G., Yu H., Chen L., Liu D. Model based Byzantine fault detection technique for cloud computing. Proceedings of the 2012 IEEE Asia-Pacific Services Computing Conference.

[12] Postel J. Internet Control Message Protocol, STD 5, RFC 792, 1981. https://www.rfc-editor.org/info/rfc792.

[13] Tajiki M.M., Shojafar M., Akbari B., Salsano S., Conti M., Singhal M. (2019). Joint failure recovery, fault prevention, and energy-efficient resource management for real-time SFC in fog-supported SDN. Comput. Netw..

[14] Cheng C.-Y., Su Z.-J., Chen C.-C., Chen S.-J., Wang W.-J. Supporting software-defined HA clusters on OpenStack platform. Proceedings of the 2017 International Conference on Applied System Innovation (ICASI).

[15] Lee Y.-L., Ho M.-H., Suharsono A., Pan Y.-C., Wang W.-J., Liang D. NCU-HA: A lightweight HA system for kernel-based virtual machine. Proceedings of the 2017 International Conference on Platform Technology and Service (PlatCon).

[16] Tchana A., Broto L., Hagimont D. Approaches to cloud computing fault tolerance. Proceedings of the 2012 International Conference on Computer, Information and Telecommunication Systems (CITS).

[17] Gama E.S., Immich R., Bittencourt L.F. Towards a Multi-Tier Fog/Cloud Architecture for Video Streaming. Proceedings of the 2018 IEEE/ACM International Conference on Utility and Cloud Computing Companion (UCC Companion).

[18] Wu Y., Yuan Y., Yang G., Zheng W. (2009). An adaptive task-level fault-tolerant approach to grid. J. Supercomput..

[19] Trihinas D., Pallis G., Dikaiakos M. (2015). Monitoring elastically adaptive multi-cloud services. IEEE Trans. Cloud Comput..

[20] Intel, Hewlett-Packard, NEC, Dell Intelligent Platform Management Interface Specification v2.0. https://www.intel.com/content/dam/www/public/us/en/documents/product-briefs/ipmi-second-gen-interface-spec-v2-rev1-1.pdf.

[21] Rahman M.S., Uddin M.Y.S., Hasan T., Rahman M.S., Kaykobad M. (2017). Using adaptive heartbeat rate on long-lived TCP connections. IEEE/ACM Trans. Netw..

[22] Gao Z., Cecati C., Ding S.X. (2015). A survey of fault diagnosis and fault-tolerant techniques—Part I: Fault diagnosis with model-based and signal-based approaches. IEEE Trans. Ind. Electron..

[23] Gao Z., Cecati C., Ding S.X. (2015). A survey of fault diagnosis and fault-tolerant techniques—Part II: Fault diagnosis with knowledge-based and hybrid/active approaches. IEEE Trans. Ind. Electron..

[24] Yu M., Wang D. (2013). Model-based health monitoring for a vehicle steering system with multiple faults of unknown types. IEEE Trans. Ind. Electron..

[25] Zhang X., Tang L., Decastro J. (2012). Robust fault diagnosis of aircraft engines: A nonlinear adaptive estimation-based approach. IEEE Trans. Control Syst. Technol..

[26] Bezerra C.G., Costa B.S.J., Guedes L.A., Angelov P.P. (2016). An evolving approach to unsupervised and real-time fault detection in industrial processes. Expert Syst. Appl..

[27] Sematext Sematext Monitoring. https://sematext.com/docs/monitoring/#setting-up-monitoring-agents.

[28] AppDynamics Overview of End User Monitoring. https://docs.appdynamics.com/display/PRO45/Overview+of+End+User+Monitoring.

[29] Datadog Synthetic Monitoring. https://docs.datadoghq.com/synthetics/.

[30] Goto Y. (2011). Kernel-based virtual machine technology. Fujitsu Sci. Tech. J..

[31] Ashley W.D. (2019). Foundations of Libvirt Development.

[32] Khan M., Toeroe M., Khendek F. Comparing Pacemaker with OpenSAF for Availability Management in the Cloud. Proceedings of the 2017 IEEE International Conference on Edge Computing (EDGE).

[33] Aviziens A. (1976). Fault-Tolerant Systems. IEEE Trans. Comput..

[34] Isermann R. (2017). Supervision, fault-detection and fault-diagnosis methods—A short introduction. Combustion Engine Diagnosis: Model-Based Condition Monitoring of Gasoline and Diesel Engines and Their Components.

[35] Zhao F., Koutsoukos X., Haussecker H., Reich J., Cheung P. (2005). Monitoring and fault diagnosis of hybrid systems. IEEE Trans. Syst. Man Cybern. Part B Cybern..

[36] Lu C.-D. Scalable Diskless Checkpointing For Large Parallel Systems. https://www.ideals.illinois.edu/bitstream/handle/2142/11054/Scalable%20Diskless%20Checkpointing%20for%20Large%20Parallel%20Systems.pdf?sequence=2&isAllowed=y.

